# Medical Legislation

**Published:** 1888-01

**Authors:** 


### MEDICAL LEGISLATION.

The efforts that, during the last few years, have been made to bring about sanitary reforms, to prevent sickness, have very naturally suggested the desirability of also securing for those who can not or do not escape sickness, a greater degree of knowledge and skill on the part of physicians and surgeons — a higher grade of qualification for the management of disease itself. In several States laws have been in operation for several years; in others tentative efforts are now being made to regulate the practice of medicine. The need of some such regulation is very generally recognized by the non-professional, as well as by the professional, public; in fact, by almost everybody except the quacks. How best to meet this need of society is, however, not quite so evident. Different plans are suggested; in different States diverse laws have been enacted, all, however, seeking to secure better education and good character, while not attempting to say what drugs or methods shall be used, but leaving those who give evidence of a knowledge of the great underlying facts and principles of medicine quite free in the use and application of those facts and principles. In other words, not attempting to decide when doctors disagree, but only requiring that all who practice medicine shall be doctors — not in title only, but in fact, learned in the science and skilled in the art of medicine. Absolute uniformity in practice would evidently be impossible, even among the graduates of a single college. Only well-established facts are uniform and permanent; opinions, even though plausible, are various and fluctuating.

How shall a State secure for its inhabitants the best skill? How protect them from imposture and ignorance? Of the three things desirable — 1, natural ability; 2, good education; 3, good character, or honesty in the use of knowledge — it is evident that we can not always secure the first; can not prevent a fool from studying medicine any more than we can keep him out of the schools of law or of theology, and to such an one learning brings but little added strength. To the student who sought to recommend himself to the learned Erasmus by saying *decem annos consumpsi in legendo Cicerone*, the scholar only replied by repeating "*one*," the vocative of the Greek word for ass. No ten years will make of one, naturally deficient, a strong, useful member of the profession, and we can not, however desirable it might be to do so, prevent such from studying; nor can we prevent them from graduating, nor from practicing, nor from getting such recognition as their knowledge may enable them to secure. It is manifestly impossible to select as students those naturally gifted or naturally apt for medical study and practice.

Over the second and third qualifications, however, the public can exercise some control. The methods by which this can be best accomplished are as yet matters of uncertainty. Our most learned men and most careful observers are by no means agreed as to the ways and means. This difference in opinion seems to depend, in part, at least, upon the tendency to look at only one side of the question. Some say: "All doctors ought to be learned, and, therefore, any law which does not secure this is a failure." From this standpoint there is abundant ground for criticism, not only of all existing laws, but, we apprehend, of all that shall be enacted. Is there any way, however, of bettering the qualifications of the profession, even though we do not accomplish all that we desire? Most certainly there is. In some of the States this betterment has already been reached, though much more can probably be done. To do this, however, we must deal with things as we find them, not as we imagine they ought to be.

It is manifestly impossible for the State to say that any one, no matter how ignorant, shall not study medicine. It is impossible for the State to determine the conditions upon which medical schools may admit or matriculate pupils. To attempt to do this is to attempt what is impracticable. The State may have a right, when any one proposes to practice medicine, to inquire into his or her knowledge of medical facts and as to his or her personal character, just as it may into the legal knowledge and character of those proposing to practice law. It is practicable to fix a standard of attainment in some way, or authorize some authority to, in some way, do this. There are two methods or modes of procedure open. One is to carefully investigate the length and breadth of the curriculum and the methods and the thoroughness of the instruction in the different medical schools, and permit to practice in the State only the graduates of such schools as, in their requirements for admission and for their degrees, reach a fair average standard; the diploma of such schools, together with the evidence of good character, being received as *prima-facie* evidence of qualification. This is the course adopted, with a good degree of success, in Illinois. The other method is to require every person proposing to practice medicine to pass an examination before a properly and legally appointed board, and to prove himself qualified to enter upon the practice of the profession. This is the case in Minnesota. Of course, there can be no absolute standard of qualification and no uniform standard in different States, but the effort should be made to secure for a community a sufficient number of medical men with the highest possible qualifications.

That there are difficulties in the execution of either one of these methods is very clearly shown by the fact that good, capable men are at variance in their opinions upon the subject. We think, however, (1) that as a matter of justice the conditions upon which men are admitted to practice should be such as can be complied with by graduates of medical colleges of other States and of other countries, as well as those of the State in which the law is in force; (2) there must be some authority, board, or committee, to determine what standard of excellence or knowledge shall be required. This cannot be done by legislative enactment, for the reason that the standard will be variable and regulated by the supply and demand, just like that of any other service, as, for instance, school-teaching or the practice of law. Although the Illinois law has worked well, upon the whole, we are inclined to think that something like the Minnesota law, requiring that every one shall be examined, no matter from what country or State or from what college he or she may come, is the better plan. The college that does the best teaching serves best the State, and will find that a larger percentage of its students will pass the ordeal of the State examination.

Of course no member of any teaching institution should be a member of the examining board. Many minor details must require careful thought, and after all there will be friction and there must be dissatisfaction. We must remember that all efforts in this direction are still tentative, and that though upon high Olympus Minerva came in the fullness of her intellectual vigor, as well as personal beauty, from the brain of Jove, such things seldom happen among mortals.

The possibility of some unifom regulations in the different States has been discussed, and, at the last meeting of the American Medical Association, a committee was appointed, with instructions to prepare a plan or draft a law which might meet the views of those interested in the different States. Whether uniformity of legislation is possible or not, it is, nevertheless, worth while to consider the question in all its bearings. For ourselves, we confess that it does not seem probable that the members of the legislatures of two or more States can be brought to think alike, or so nearly alike as to adopt the same means and in the same language, nor do we think this is a matter of as much consequence as do some of our friends in the profession. It is, however, desirable that, in some way, neighboring States shall be able to require something like uniform qualifications on the part of those who are permitted to practice; otherwise, the more incompetent will gravitate to those States where the standard is lowest. We have seen something like this as a result of the enforcement of the Illinois law. Many hundreds, who could not comply with the law, emigrated to Michigan, Indiana, Missouri, Iowa and Wisconsin. Like wolves and bears, they are now being pressed still further towards the border-land of civilization.

